# Association between coronavirus cases and seasonal climatic variables in Mediterranean European Region, evidence by panel data regression

**DOI:** 10.1007/s13762-021-03698-0

**Published:** 2021-10-13

**Authors:** A. R. M. Alsayed

**Affiliations:** Department of Economics, Management and Quantitative Methods, University of Milan, 20122 Milan, Italy

**Keywords:** Coronavirus pandemic, Panel data analysis, Prediction, Seasonal effect, Temperature, Wind speed

## Abstract

The coronavirus pandemic is one of the most fast-spreading diseases in the history, and the transmission of this virus has crossed rapidly over the whole world. In this study, we intend to detect the effect of temperature, precipitation, and wind speed on the Coronavirus infected cases throughout climate seasons for the whole year of epidemic starting from February 20, 2020 to February 19, 2021 with considering data patterns of each season separately; winter, spring, summer, autumn, in Mediterranean European regions, whereas those are located at the similar temperature zone in southern Europe. We apply the panel data approach by considering the developed robust estimation of clustered standard error which leads to achieving high forecasting accuracy. The main finding supports that temperature and wind speed have significant influence in reducing the Coronavirus cases at the beginning of this epidemic particularly in the first-winter, spring, and early summer, but they have very weak effects in the autumn and second-winter. Therefore, it is important to take into account the changes throughout seasons, and to consider other indirect factors which influence the virus transmission. This finding could lead to significant contributions to policymakers in European Union and European Commission Environment to limit the Coronavirus transmissions. As the Mediterranean region becomes more crowded for tourism purposes particularly in the summer season.

## Introduction

The coronavirus pandemic (COVID-19) is one of the most historical deadly contagions. The SARS of COVID-19 was identified in the city of Wuhan, Hubei province, China, at the end of the year 2019, thereafter it has been spreading across the worldwide within few months, and thus, it became a resultant disease and causing a huge number of deaths, as well serious infections. Recently, the World Health Organization (WHO 2020) declared that the COVID-19 SARS is a serious pandemic and probably it remains to threaten for the long term, while most of the population will mild to moderate respiratory illness and recover without requiring special treatment. On the other hand, scientific research proved that increasing the spread of epidemics could be partially associated with some factors or exogenous variables, some of those are the environment quality, pollution, and climatic variables such as the air temperature and humidity, ecological system, the social environment, and population density (Alsayed and Manzi 2018; Zaidi et al. 2015). Moreover, medical researches provided that respiratory system diseases such as influenza virus and SARS infections are more common to happen in colder environmental regions, while it could be decreased at higher climatic temperatures. One of the previous research pointed out that the SARS infection was 18 times higher at lower air temperatures than at higher temperatures (Lin et al. 2006). In addition, Park et al. (2020) identified that the climatic factors can significantly affect the viral transmission of SARS of COVID-19, as it increases significantly with low temperatures and high relative humidity.

Recently, several types of research are being developed due to the importance of understanding the COVID-19 outbreak and the association with the meteorological factors by using various statistical techniques at global level or local level. In the aspect of global level, Roy (2020) explored the association between the global temperature into spreading the COVID-19, during March and April 2020. The countries are categorized into four different categories based on the variability of their temperature level. The findings suggest that the different temperature categories can influence virus transmission variously. Ahmed and Ghanem (2020) detected the influences of the environmental conditions such as temperature, humidity, precipitation, wind, and air pollution, in spreading the COVID-19 virus, besides other factors related to behaviour of people such as smoking, drinking wine, and eating pig meat. The results exhibited a significant relationship between the suggested conditions and the virus spread. Such high rate of virus spread linked to area having humidity levels ranged between humid and dry-sub humid. On the other side, in the aspect of local level, Ali et al. (2021) investigated the impact of wind speed and air pollution on COVID‑19 transmission for the period March 10, 2020, until October 4, 2020 in Pakistan. The findings support that wind speed has a positive correlation with COVID-19 transmission in most provinces, while there is inverted U-shaped relationship between wind speed and COVID-19 transmission in Punjab province.  Also, there is inverted U-shaped between particulate matter and COVID-19 transmission. The inverted U-shaped shows a positive relationship at the beginning stage, then becomes downward after a certain peak (Alsayed and Sek 2013; Isa et al. 2015). An additional, using advance statistical techniques to study environmental variables could provide more accurate estimation models. Alsayed and Manzi (2019) detect the relationship between Carbon dioxide emissions as proxy of environmental degradation with other associated factors using the developed Monotonic Dependence Coefficient verses the classical monotonic correlation measures such Pearson’s r, Spearman’s ρ and Kendall’s τ. The findings provided that the developed Monotonic Dependence Coefficient perform better in some cases. Al Sayed et al. (2018) considered several diagnostic statistical methods to detect the outliers, leverages and the influence points for extreme values of the environmental variables of panel data. Zaidi et al. (2017) applied an inversed function regression to minimize the error term of the estimation model.

Nevertheless, the impact of this pandemic is still highly uncertain and thus, in this setting, it would be more interesting to investigate the related factors of spreading the COVID-19 in-depth study, and to point the role of ambient temperature on the survival and transmission of COVID-19 viruses. Therefore, the prediction of the confirmed and death cases are important to be considered in policies, as it can help the public health system to develop a strategic plan to deals with the outcome of COVID-19 and to be more ready for healthcare efficiency and to avoid overcrowding in hospitals and preventing new deaths. Also, it is informative for policymakers at government level to make assertively decision to control the transmission of the epidemic. For that purpose, the current study intends to detect association between COVID-19 infected cases and climatic variables, namely; average temperature, minimum and maximum temperature (°C), precipitation (mm), and average wind speed (m/s) for the whole year of epidemic with considering each season separately; winter, spring, summer, autumn, in the Mediterranean European regions where located at the similar temperature zone in southern Europe.

The contributions of this study are to cover large datasets for the whole year of the COVID-19 pandemic; February 20, 2020 till February 19, 2021 with including more features associated with weather and climatic conditions, then modelling those data according to the climatic seasons to examine properly the seasonal data patterns on short term and long term by applying panel data approach with considering all the diagnostic assumption to detect the effect of each input variable for each country, also applying robust estimation clustered standard error which leads to achieve high forecasting accuracy. Moreover, to detect the changing of the infected cases throughout climate seasons which could lead to significant findings to policymakers in European Union and European Commission Environment. To reach that, several models are estimated to understand the dynamic of data as illustrated in Fig. 2.

## Materials and methods

In this section, we illustrate the dataset features and the statistical approaches with the analysis process.

### Data description

This study focuses on the region of Mediterranean Countries in the European Union, which occupied 20.6% of EU territory and consists of seven member countries either completely; Greece, Malta and, Cyprus, or partially encompasses France, Portugal, Italy, and Spain. The Mediterranean basin stretches about 3800 km from the east the strait of Portugal to the west the shores of Lebanon, while the width ranges around 1000 km from north Italy to the south of Libya shores. The climate of Mediterranean region is dry hot in summer, but humid and cool in winter, whilst, it might be capricious with sudden torrential rainfall or bouts of high winds during the year. Those various climatic conditions notoriously have a profound influence on several aspects such as the vegetation, industrials, trading of the region, and the spread of the epidemic. Moreover, the topography of the European Mediterranean region has various features or landscape; high mountains, impenetrable scrub, semi-arid steppes, coastal wetlands, rocky shores, sandy beaches and clear sea, which might affect differently the epidemic transmission (European Environment Agency 2009). Further, the period of the four seasons follows one another regularly; winter, spring, summer, and autumn, generally begin at 21st of December, 21st of March, 21st of June and 22nd of September, respectively.

**Fig. 1:**
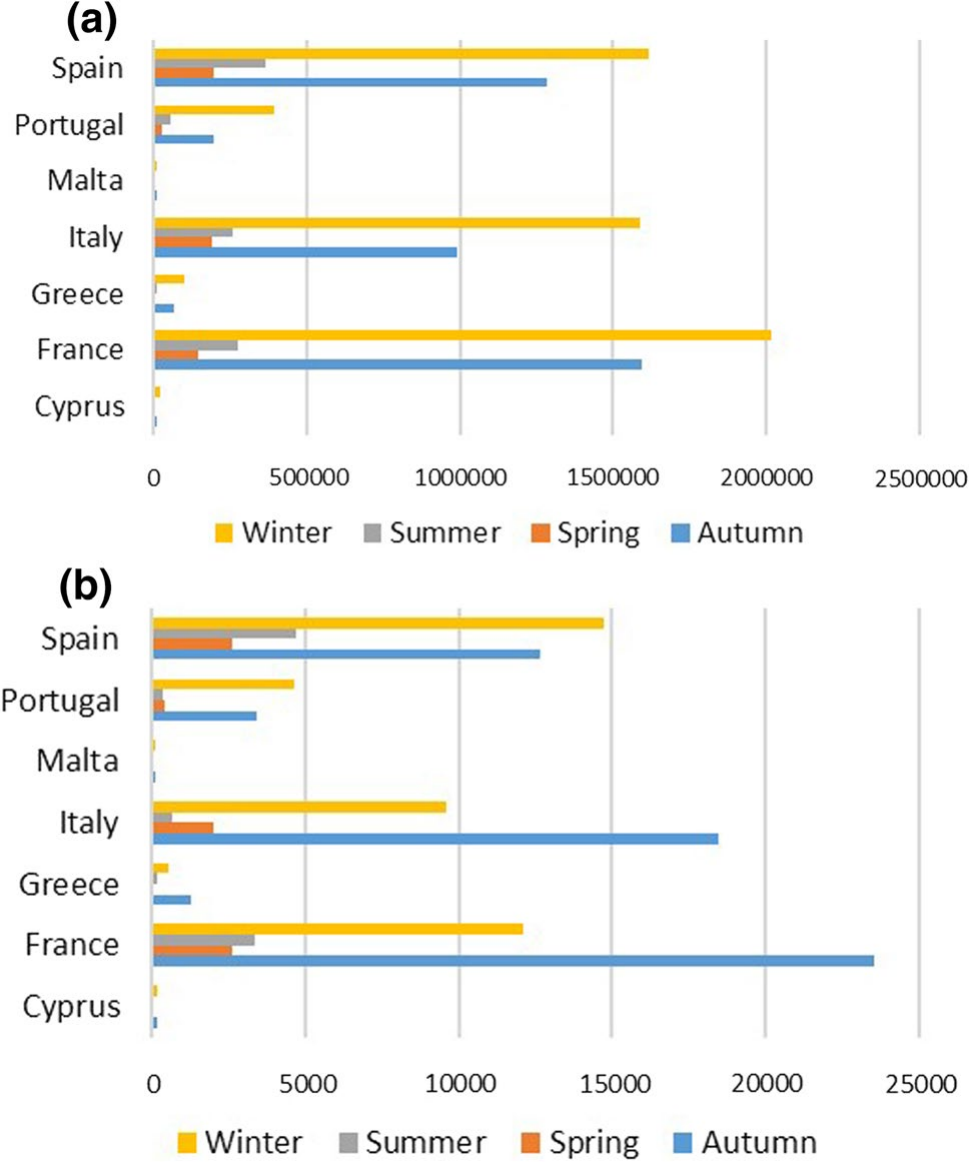
**a** The total infected cases per country, **b** the new infected cases per country

The data are collected from the beginning of the epidemic in daily form for a time span from February 20, 2020 till February 19, 2021 for European Union Mediterranean Countries. The daily COVID-19 data were extracted from the Our World in Data website, while the meteorological variables are retrieved from the NASA-POWER website which are selected from the nearest monitoring stations to each country's capital, that because the capital cities mostly crowded and cover most of the local and international movements, also it includes the economic-social activities. Further, the dependent variable is the infected COVID-19 cases, while the independent variables are average temperature, minimum and maximum temperature at two metres above the surface which measured by Celsius (°C) degree, wind speed range at 50 m (m/s), and precipitation per inches (mm). Finally, the dataset is combined and reshaped in conformity with conventional panel data analysis. The summary of the descriptive statistics of each country illustrated in Table 1. Moreover, Fig. 1a, and b provide the total infected cases and new infected cases for each season in each country, respectively. It is obvious that the highest total infected cases in France during the winter, also the highest number of new infected cases is in France but during the autumn.

**Table 1:** Descriptive statistics

Country	Season	*n*	Total infected cases	New infected cases	Average temperature	Precipitation	Wind speed
Cyprus	Autumn	90	6880	182	5.70	5.67	8.66
	Spring	92	762	9	7.38	2.55	6.91
	Summer	93	1248	7	7.34	4.19	7.61
	Winter	91	18,483	166	7.07	4.51	9.67
France	Autumn	90	1,595,774	23,541	7.06	1.24	3.07
	Spring	92	146,517	2631	6.69	1.00	2.68
	Summer	93	276,029	3327	6.15	0.89	2.88
	Winter	91	2,012,947	12,077	6.80	2.56	2.98
Greece	Autumn	90	63,716	1285	7.80	1.37	4.38
	Spring	92	2445	29	6.58	1.32	4.04
	Summer	93	7086	136	7.22	1.59	4.21
	Winter	91	100,298	510	8.20	1.75	4.32
Italy	Autumn	90	988,462	18,479	6.03	1.64	2.27
	Spring	92	191,651	2013	5.03	0.68	1.61
	Summer	93	257,178	670	5.43	1.17	1.99
	Winter	91	1,587,271	9564	5.89	0.95	1.98
Malta	Autumn	90	7040	99	7.76	1.96	6.14
	Spring	92	465	6	8.26	1.25	5.54
	Summer	93	1292	24	7.19	2.09	5.79
	Winter	91	10,605	97	7.76	1.79	6.06
Portugal	Autumn	90	193,880	3406	7.10	1.26	5.13
	Spring	92	24,124	414	7.79	1.53	4.72
	Summer	93	52,798	328	6.73	1.69	4.92
	Winter	91	391,515	4613	7.67	1.81	5.07
Spain	Autumn	90	1,284,497	12,633	7.04	1.37	3.73
	Spring	92	196,233	2627	5.79	1.12	3.42
	Summer	93	365,891	4688	7.06	1.37	3.58
	Winter	91	1,616,293	14,716	6.99	1.21	3.66

The total observations are 2562, while the number of observation for each country is 366

**Fig. 2:**
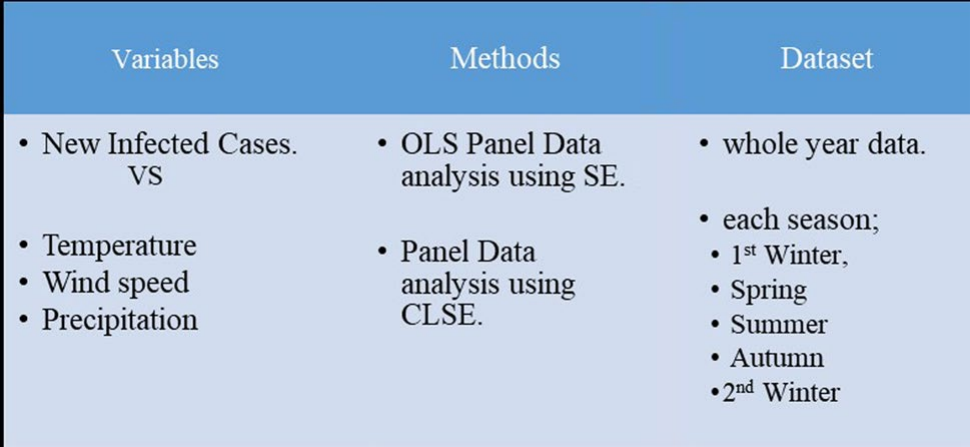
Flow chart shows the process of analysis

### Statistical approaches

In this section, we illustrate the panel data regression which is used to investigate the real effect of the climatic variables on the spread of COVID-19 for the selected countries in this study. This approach is used as it is the most suitable technique to detect the effect in panel countries and each individual country, and thus, it leads to accurate prediction of COVID-19 transmission.

The panel data analysis is a combination of longitudinal data observed by countries over a period of time. The advantage of applying that is to control the variation of time series and cross sections simultaneously, and to detect the presence of unobserved heterogeneity as it includes a large number of data and it increases the degree of freedom but reduces the collinearity. Hence improve the efficiency of estimates and broaden the scope of inference. It could illustrate the correlation within-country and between countries. The Process of analysis to obtain the most accurate model starts by estimating the pooled model, fixed effects models within, between model, and random effects model, then deciding between random and fixed by using Hausman test which tests whether the errors are correlated with the regressors, then deciding between fixed and pooled model by using redundant test, final step is to estimate the robust model by applying the developed clustered standard errors estimation (Baltagi 2021).

In addition to that, to obtain accurate estimation models, we need to check the assumptions of the panel data regression by the diagnostic analysis such as test for cross-sectional dependence, serial correlation or autocorrelation, and heteroscedasticity of the residuals. The violation of those assumptions leads to bias results, particularly for a long time series period (Baltagi 2008). Cross-sectional dependence can be checked by using both the Breusch-Pagan LM test of independence and the Pasaran CD test, the null hypotheses of those tests illustrate that there is no cross-sectional dependence as the residuals across entities are not correlated. Whilst, the serial correlation in panel models can be examined by the Breusch-Godfrey or Wooldridge test, the null hypotheses indicate that there is no serial correlation. Whereas, the stationarity or unit-roots could be detected by the Dickey-Fuller test as it checks the stochastic trends of the data series, the null hypothesis denotes that the series has a unit root or non-stationary trend. Further, we use the Breusch-Pagan test to check the heteroscedasticity, the null hypothesis for the test shows that the data are homoscedasticity.

Generally, the autocorrelation and heteroscedasticity are almost certain particularly in panel data due to existing of the correlation between the errors within individual essentially, persistent effects through time, thus the conventional estimates of the standard errors are incorrect, so it is important to apply an accurate tool to consider the effects of the residuals (Alsayed et al. 2020). There are several methods to calculate the standard errors for regression procedures, such as robust standard errors or white method, but some recent studies have proved that those methods are inconsistent in the case of the panel fixed-effects regression model, and the standard errors for the existence of correlation across time-periods within the entities can be corrected by using clustered standard errors (Stock and Watson 2008). To tackle that problems and avoiding the bias estimation, we apply the common robust covariance matrix or Sandwich estimator (SE), and the developed cluster-standard errors estimations (CLSE), as the standard error gets smaller by clustering and it makes the models achieving high forecasting accuracy.

Nonetheless, the hypothesis we assumed in this study that the spread of the COVID-19 virus will decrease in the countries with a higher temperature than countries with lower records but with different rates according to the climatic seasons. Further, the contemporary disease is spreading exponentially around the world, so the dependent variable will be log-transformed to follow the normal distribution as the original data are skewed highly in the selected countries. The interested estimating model of the panel data is illustrated in Eq. 1.

1 $$\begin{aligned} \log (CIC_{{{\text{it}}}} ) &= \alpha_{i} \, + \, \beta_{1} at_{{{\text{it}}}} + \beta_{2} mit_{{{\text{it}}}} + \beta_{3} mxt_{{{\text{it}}}}\\& \quad + \beta_{4} {\text{ ws}}_{{{\text{it}}}} + \beta_{5} {\text{ p + Dummy}}_{{{\text{it}}}} + \varepsilon_{{{\text{it}}}} \end{aligned}$$

where *i* is the country of European Mediterranean region, *t* is the day throughout the year, σ is a constant. While y is the cumulative COVID-19 infections (CIC), *β* is a vector of coefficients of the independent variables; average temperature (at), minimum (mit) and maximum temperature (mxt), wind speed (ws) and precipitation (p). ε_it_ is the error terms.

### Process of analysis

The process of analysis constructs of several stages to model the infected COVID-19 cases by applying the panel data analysis using the SE and CLSE for both whole dataset and each season separately, as shown in Fig. 2.

## Results and discussion

**Fig. 3:**
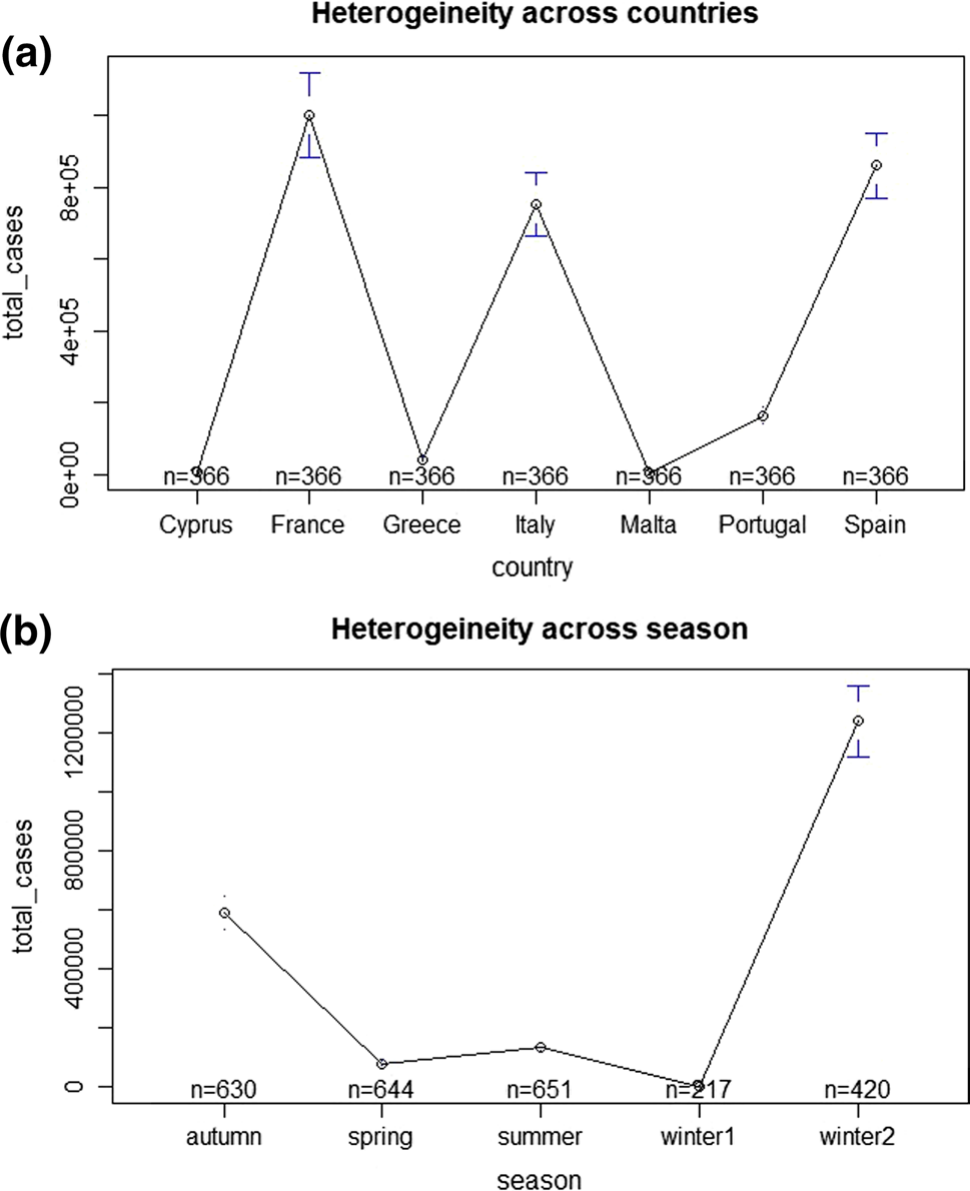
**a** Heterogeneity across countries, **b** heterogeneity across seasons

The output of testing the assumptions shows significant results of the Dickey-Fuller test which means that the series of data is stationary (no unit-root), so it is not preferred to model the data by taking the first difference of the variable. However, the output shows significant results of Breusch-Pagan LM, Pasaran CD test, and Breusch-Godfrey test, which means that there are cross-sectional dependence and serial correlation. Whilst, Breusch-Pagan test indicates the existence of heteroskedasticity, to tackle that we apply a cluster-standard errors estimations CLSE. Further, Fig. 3a shows that there is a heterogeneity of the infected COVID-19 cases among the selected countries, meaning that each country at different season has been effected differently by the COVID-19 transmission. Also, Fig. 3b illustrates that there is a heterogeneity of the infected COVID-19 cases among the climate seasons which supports that the climatic variables could affect differently according to the climate season.

**Fig. 4:**
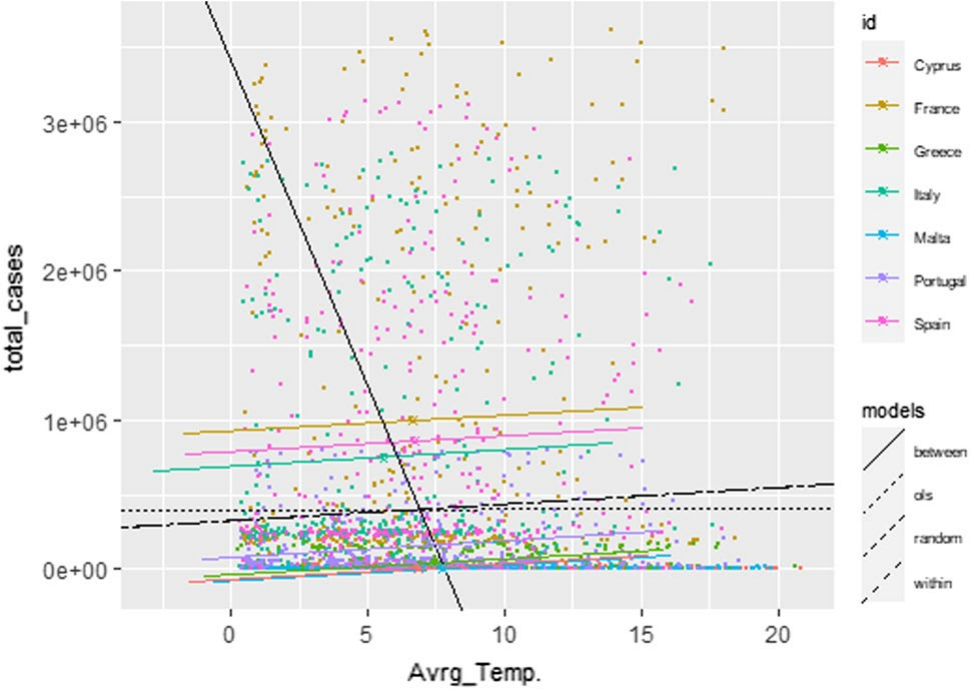
The estimated regression lines for each model

Table 2 provides the results of panel data regression for the whole dataset using CLSE. According to Lagrange Multiplier Test which shows a significant effect of the performance of the fixed-effect model within-time, as it is more robust model to consider the heterogeneity across time, as it is clear to note that from Fig. 3b. Also, it has the highest R-square value among the estimated models, thus it is more accurate to interpret the results base on this model. Also, Fig. 4 illustrates the estimated regression line of the infected cases with the average temperatures, and we can see clearly that the FE within-time are more accurate to represent the dataset. On the other hand, the insignificant variables are re-examined again but the output showed also insignificant results, that is the reason to exclude them from the final estimated models in Table 3.

**Table 2:** Estimated models of the infected cases and climatic variables by using CLSE for whole dataset

Estimated Coefficients	Pooled	FE between	FE within-time	Random effects
Average temperature	− 6.08*	− 7.36*	− 3.67**	− 3.20*
	(3.11)	(2.12)	(1.55)	(1.86)
Minimum temperature	− 6.07*	− 7.02*	− 3.18*	− 3.11*
	(3.11)	(2.12)	(1.55)	(1.87)
Maximum temperature	6.01*	7.03	− 3.24*	− 3.03*
	(3.11)	(2.12)	(1.56)	(1.87)
Average wind speed	− 0.310***	− 0.783**	− 0.415***	− 0.410***
	(0.07)	(0.35)	(0.08)	(0.09)
Precipitation	− 0.005	− 0.001	0.003	0.003
	(0.001)	(0.004)	(0.002)	(0.002)
Constant	6.16***	–	–	6.54***
	(0.34)	–	–	(0.95)
Balanced panel: *n* = 7, *T* = 366, *N* = 2562				
* R*-squared	0.20	0.27	0.68	0.64
Adj. *R*-squared	0.19	0.26	0.62	0.64
* F*-statistics	124.09***	184.65***	908.05***	4,453.6***

The numerical values are F&T-test which are significant at 1% level ***; at 5% level ** and at 10% level *. Standard error in parentheses

**Table 3:** Estimated models of the infected cases and climatic variables by using CLSE for each season separately

Estimated coefficients	1st winter	Spring	Summer	Autumn	2nd winter
Average temperature	0.006**	− 0.004	− 0.002	− 0.011	− 0.018**
	(0.003)	(0.0009)	(0.006)	(0.007)	(0.007)
Average wind speed	− 0.59***	− 0.55***	− 0.51***	− 0.43***	− 0.23***
	(0.060)	(0.08)	(0.075)	(0.066)	(0.062)
Balanced panel: *n* = 7 countries					
Observations *N*	217	644	651	630	420
* R*-squared	0.838	0.732	0.729	0.768	0.600
Adj. *R*-squared	0.810	0.692	0.682	0.729	0.532
* F*-statistic	476.006***	500.424***	497.175***	891.220***	268.991***

The numerical values are F&T-test which are significant at 1% level ***; at 5% level ** and at 10% level *. Standard error in parentheses

Generally, all estimated models in Table 2 indicate that the average temperature, maximum temperature, and wind speed have significant effects on the COVID-19 infected cases. We interpret our results based on the within-time fixed effect model as it has obtained the highest R-square value 0.68 which indicating that the model could explain or predict about 68% of the infected cases when it is interpreted based on the log-transformation in the linear model.

For every one-unit increase in Celsius degree in the average temperature, the number of infected cases decreases by approximately 3.67% cases per day within the threshold of temperature which is set as the maximum reported degree of temperature. This results in line with the (Roy 2020; Ahmed and Ghanem 2020) in the beginning of the epidemic period during first-winter and spring. Further to that, the findings support that the higher speed of the wind, could reduce the infection cases, for every one-unit of increasing in the wind-speed (m/s), the number of COVID-19 cases decreases by about 0.42%. Wind speed could affect directly or indirectly to reduce the infection transmission, which might due to social behaviour as high wind speed makes people stay more in home, so less social interaction happens, or the wind speed spreads the suspended particles in the atmosphere, which it might have a significant role in the transmissions particularly in the capitals. This finding is in line with the results of the study (Ahmed and Ghanem 2020). However, our findings show that the precipitation does not have a significant effect on the COVID-19 infected cases, which is in the same trend with finding of the study (Ahmed and Ghanem 2020).

Moreover, Table 3 shows the results for each season separately, we can note that there are various effects of temperatures among the seasons on the infected cases, whereas the estimated model of spring or summer reached up to 75% importance in the prediction of the infected cases. The increasing average temperature in first-winter and spring has significant effects to reduce the infected case more than that effect in autumn and second-winter. However, the wind speed almost has similar effects for each season, except on the second-winter. This weak relationship leads to that there are other factors which might play an important role in the effect of the various transmission particularly in the last period of this epidemic.

We conclude that the COVID-19 contagion decreases with hot and dry weather differently in each season, as the temperature and wind speed have important effect on the infected COVID-19 cases, but in autumn and second-winter season the temperature may are not the main factor and it has indirect effects into the COVID-19 transmission. Furthermore, it is very important to note that when there are more hours of sunlight, there are more people going outside for social activities with interaction, which might be a reason to increase the risk of virus transmission among people. The cultural behaviour during various climate seasons of people living in the urban area unlike those who living in a rural area, also the density is dissimilar. Thus, according to that, we suggest the social factors together with the climate variables could play important role in the effect on the spread of the COVID-19 virus differently based on climate seasons.

## Conclusion

The COVID-19 epidemic is a widespread infectious epidemic that has affected millions of people worldwide since the end of December 2019. This research intends to contribute to global community and particularly to policymakers of European Union organization to have a better understanding and a clear prediction of the coming summer season for tourism to take the appropriate measurements in the Mediterranean regions which are targeted in this study. The contribution of this study is to examine the influence of climatic variables on the spread of the virus in each season by applying accurate statistical techniques panel data analysis to consider the time effects together with developed cluster stander error estimation to improve the accuracy of predicting. It is observed that climatic variables, such as temperature and wind speed have significant influence in reducing the COVID-19 cases in the beginning on this epidemic particularly, first-winter, spring and summer, but it has very week effects in the autumn and second-winter.Therefore it is important to take into a count the changing throughout seasons; winter, spring, summer, autumn as it have various effects on the number of infected cases. According to that, we intend to extend this research to study the social effects together with the climatic variables by applying various developed statistical techniques. Finally, our model could be implemented in planning actions and policy makers to improve the health system to management of COVID-19 spreads. The study depicts that governments have to take important strict decisions to control the spread for the coming seasons.

## Data Availability

All data used are openly available and relevant websites are mentioned.
